# Unmarried Women

**Published:** 1887-08-13

**Authors:** 


### UNMARRIED WOMEN.

Is woman inferior, superior, or equal to man? The question is unanswerable and futile. Is she able to stand alone and fight her own battles in life? Here a practical and not a merely speculative issue is raised, and an answer is easy. As the question is practical, so must the answer be, and the appeal is therefore to experience. Has one woman ever stood alone and fought her own battles? Have thousands of women, from Queens and Empresses downwards, with dauntless courage and steadfast heart maintained their own ground in the sternest of life's conflicts, and against the fiercest of fate's assaults? The reply is unhesitating, unquestionable, and decisive. When women *must*, they *can* hold their own against the opposing forces of nature, and prove that they too belong to that class of beings who are placed at the head of the existing order. This no practical and experienced person for a moment doubts.

It seems certain that, in England at any rate, the number of unmarried women is rapidly increasing, and is likely to further increase. The conditions of society at present are not favourable to marriage. Women are not trained to be efficient and economical at home. Domestic duties are despised by mothers and daughters, and calls, amusements, and pleasure are looked upon as the natural things to expect both before and after marriage. We are not railing or writing sensationally—far from it. Anyone who has eyes to see with, cannot fail to perceive that among the upper and middle classes the young woman who secures neither wealth nor position by marriage, is looked upon as a complete failure. It is true she may have married a philosopher, a hero, or a saint, but that is absolutely nothing. Most of the mothers and daughters of the class under consideration are utterly destitute of the capacity for understanding anything but the gross and palpable prosperity which appeals to the eye and the pocket. As they have nothing of the philosophic, the heroic, or the saintly in their own characters, so they have not the sense to value these qualities in others.

But all men cannot be rich or titled. Sir Gorgius Midas is happily a rare person, and dukes and counts are not yet, with us at any rate, quite so thick as rabbits in a distant and protected warren. If mothers and daughters are to continue as they are, marriages will become more and more difficult, and more and more remote, and by consequence the number of unmarried women will rapidly increase. What are these women going to do? Are they going to keep an aged and overworked father with his nose to the grindstone until he drops dead from sheer exhaustion and despair? Are they going to cripple amiable and generous brothers through all the earlier and later years of their life-struggle, and to make it impossible for them to marry too? These questions are pertinent and practical, and demand to be answered.

If women fear to face life with a husband of merely moderate means, they will have to learn to face it alone and on their own account. Many of them are quite prepared for this, but unhappily these are the very women who would be willing to face it as the wives of poor men if the favourable opportunity should occur. It is not the capable and the generous-hearted who refuse to marry men who are not rich, but the vain, the frivolous, the silly, and the incapable. That is what constitutes the difficulty of the problem. What are we going to do with the hundreds of thousands of young women who have been bred to bad French, worse music, and no arithmetic at all: who dance and play tennis indifferently, and have not the least idea when the dumpling is on the table how the apple could possibly have got into it? We say what are *we* going to do with them, but we should rather ask—What are they going to do with themselves? A very cynical philosopher suggests that just as young men should be put under barrels at sixteen, and not allowed to emerge until they are five-and-twenty, and have had the angles of their conceit rounded off, so should superfluous young women, who have not been trained to do anything useful in the world, be drowned like kittens when their natural protectors are no longer able to stand between them and destitution. Having some remnants of old-fashioned philosophy about us, we are not yet prepared to go to quite such lengths as these.

But what then can be done? Can our plans be reformed? The first thing to do is to recognise the facts. The ostrich-like proceeding of burying our heads in the sand and imagining we are escaping from the hunters, will be of no avail. There are now a good many very clever women who constitute themselves the advocates of what are called "Women's Rights." Here is a commonplace but urgent problem for their solution. What can they, and what will they do for their badly trained and incapable sisters? The question is essentially one for women, and especially for those women who see and deplore the melancholy and helpless condition of so many of their sex. The really practical thing to do is to create a strong public opinion among women themselves in favour of a totally different ideal of young womanhood from that which prevails at the present time. Instead of the would-be artistic and simpering misses who now set the fashion among their sex, let us have some robust and useful young women who, if they find themselves without aptitudes for study, teaching, clerical, or literary work, can make shirts, concoct a beefsteak-pie, instruct their younger sisters in arithmetic and history, or the kitchenmaid how to scour a pan. There is a prompt necessity for root-and-branch reform. The present system of bringing up girls among the so called educated classes is laughable in its utter absurdity. Whatever be the theory, in practice it comes to this, that the vast majority of young women of the wealthier sort live for no other purpose but to dress and amuse themselves. Do they then really suppose that Almighty God created them for such a contemptible end as that? Conditions of space prevent our pursuing the subject further, but we seriously urge upon the more intelligent and purpose-like of women the immediate necessity of setting up a propaganda for the diffusion and teaching of those ideas and practical arts, endowed with which women are the salt of the household and the builders of the family fortunes, but being destitute of them they are often the ruin and destruction of the fairest prospects and the most promising lives. Vain mothers and weak fathers are more responsible for the existing state of things than daughters, and it is they who must be brought to see their mad folly before any amendment can reasonably be looked for.

